# Efficacy and Safety of Empagliflozin in Type 2 Diabetes Mellitus Saudi Patients as Add-On to Antidiabetic Therapy: A Prospective, Open-Label, Observational Study

**DOI:** 10.3390/jcm11164769

**Published:** 2022-08-16

**Authors:** Fahad M. Althobaiti, Safaa M. Alsanosi, Alaa H. Falemban, Abdullah R. Alzahrani, Salma A. Fataha, Sara O. Salih, Ali M. Alrumaih, Khalid N. Alotaibi, Hazim M. Althobaiti, Saeed S. Al-Ghamdi, Nahla Ayoub

**Affiliations:** 1 Department of Pharmacology and Toxicology, Faculty of Medicine, Umm Al-Qura University (UQU), Makkah 21955, Saudi Arabia; 2 Department of Endocrine and Diabetes, Armed Forces Hospital, Southern Region, Khamis Mushait 61961, Saudi Arabia; 3 Saudi Toxicology Society, Umm Al-Qura University (UQU), Makkah 21955, Saudi Arabia; 4 Institute of Cardiovascular and Medical Sciences, University of Glasgow, Glasgow G12 8QQ, UK; 5 Ministry of Defence, Riyadh 11159, Saudi Arabia; 6 King Faisal Medical City, Taif 26514, Saudi Arabia; 7 Department of Pharmacognosy, Faculty of Pharmacy, Ain-Shams University, Cairo 11566, Egypt

**Keywords:** empagliflozin, safety, efficacy, Saudi patients, type 2 diabetes mellitus

## Abstract

The Saudi Food and Drug Authority (SFDA) approved sodium-glucose cotransporter-2 (SGLT2) inhibitors in 2018. The efficacy and safety of empagliflozin (EMPA) have been confirmed in the U.S., Europe, and Japan for patients with type 2 diabetes mellitus (T2DM); however, analogous evidence is lacking for Saudi T2DM patients. Therefore, the current study aimed to assess the efficacy and safety of EMPA in Saudi patients (*n* = 256) with T2DM. This is a 12-week prospective, open-label, observational study. Adult Saudi patients with T2DM who had not been treated with EMPA before enrolment were eligible. The exclusion criteria included T2DM patients less than 18 years of age, adults with type one diabetes, pregnant women, paediatric population. The results related to efficacy included a significant decrease in haemoglobin A1c (HbA1c) (adjusted mean difference −0.93% [95% confidence interval (CI) −0.32, −1.54]), significant improvements in fasting plasma glucose (FPG) (−2.28 mmol/L [95% CI −2.81, −1.75]), and a reduction in body weight (−0.874 kg [95% CI −4.36, −6.10]) following the administration of 25 mg of EMPA once daily as an add-on to ongoing antidiabetic therapy after 12 weeks. The primary safety endpoints were the change in the mean blood pressure (BP) values, which indicated significantly reduced systolic and diastolic BP (−3.85 mmHg [95% CI −6.81, −0.88] and −0.06 mmHg [95% CI −0.81, −0.88], respectively) and pulse rate (−1.18 [95% CI −0.79, −3.15]). In addition, kidney function was improved, with a significant reduction in the urine albumin/creatinine ratio (UACR) (−1.76 mg/g [95% CI −1.07, −34.25]) and a significant increase in the estimated glomerular filtration rate (eGFR) (3.54 mL/min/1.73 m^2^ [95% CI 2.78, 9.87]). Furthermore, EMPA reduced aminotransferases (ALT) in a pattern (reduction in ALT > AST). The adjusted mean difference in the change in ALT was −2.36 U/L [95% CI −1.031, −3.69], while it was −1.26 U/L [95% CI −0.3811, −2.357] for AST and −1.98 U/L [95% CI −0.44, −3.49] for GGT. Moreover, in the EMPA group, serum high-density lipoprotein (HDL) significantly increased (0.29 mmol/L [95% CI 0.74, 0.15]), whereas a nonsignificant increase was seen in low-density lipoprotein (LDL) (0.01 mmol/L [95% CI 0.19, 0.18]) along with a significant reduction in plasma triglyceride (TG) levels (−0.43 mmol/L [95% CI −0.31, −1.17]). Empagliflozin once daily is an efficacious and tolerable strategy for treating Saudi patients with insufficiently controlled T2DM as an add-on to ongoing antidiabetic therapy.

## 1. Introduction

The growing burden of type 2 diabetes mellites (T2DM) is a crucial issue in health care worldwide. T2DM continues to increase in prevalence and incidence and is a significant cause of human suffering and death. Despite sizable investments in clinical care, research, and public health interventions, there appears to be no signal of reduction in the rate of disease increase [1]. According to the World Health Organisation (WHO), Saudi Arabia has the second highest diabetes prevalence of all Middle Eastern countries (7th in the world), with an estimated population of seven million individuals living with diabetes and more than three million with prediabetes [2,3,4]. Moreover, by 2030, this number is expected to more than double [5].

EMPA was approved by the U.S. Food and Drug Administration (FDA) in 2014 as an adjunct to diet and exercise to improve glycaemic control in adults with T2DM [6]. EMPA is a selective sodium-glucose cotransporter 2 (SGLT2) inhibitor. It is characterised by its unique mechanism as a hypoglycaemic agent. Specifically, it depends on enhancing glycosuria away from insulin independence. This unique mechanism enables EMPA to achieve controllable hypoglycaemic action [7]. Studies conducted in the USA, Canada, UK, and Japan have recommended using EMPA alone or together with other anti-diabetic agents as a cost-effective oral treatment for T2DM once daily [8]. Many clinical trials have reported the antihyperglycemic effect of EMPA due to a reduction in haemoglobin A1c (HbA1c) and fasting plasma glucose (FPG) levels [9,10,11,12]. In addition, EMPA is reported to reduce body weight, waist circumference, and body fat index in patients with T2DM [13,14].

In 2016, the U.S. FDA approved EMPA to reduce the risk of cardiovascular death in adult patients with T2DM and cardiovascular disease. Many studies have documented the relative reductions in the risk of cardiovascular death and hospitalisation with EMPA versus placebo [7,15,16,17,18]. In addition, EMPA causes significant natriuresis [19], rapid reductions in pulmonary artery pressure, and reduced LV volumes in patients with heart failure and reduced ejection fraction (HFrEF) [20,21,22]. The cellular mechanism by which EMPA improves cardiovascular outcomes is its ability to stimulate erythropoiesis via an early increase in erythropoietin production in people with T2DM [23]. Early administration of EMPA may attenuate changes in extracellular water and intracellular water (ICW) in patients with acute myocardial infarction [24].

EMPA slows the progressive decline in kidney function in patients with HFrEF, with or without diabetes [25,26]. The short- and long-term benefits of EMPA on urinary albumin excretion have also been shown [27]. In addition, the haemodynamic effects of EMPA associated with lower glomerular pressure may contribute to the long-term preservation of renal function. [28]. Furthermore, EMPA improved glycaemic control in renal transplant recipients with post-transplantation diabetes mellitus (PTDM) compared with a placebo [29]. Sattar et al. proved that EMPA reduces liver enzymes ALT and AST in patients with T2DM in a pattern consistent with a reduction in liver fat, particularly when ALT levels are high [30,31,32].

Several studies have reported the safety and efficacy of EMPA for T2DM [11,33,34,35]. EMPA was the first in class to not only demonstrate safe SGLT2 inhibition but also cardio- and reno-protective effects in an adequately powered cardiovascular outcome trial [36]. However, EMPA was associated with an increased risk of hypoglycaemia and genital and urinary tract infections [37].

The Saudi Food and Drug Authority (SFDA) approved SGLT2 inhibitors in 2018 [38]. The current SFDA-approved drugs in this class include canagliflozin, dapagliflozin, and EMPA. Based on the literature, the efficacy and safety of empagliflozin have been confirmed in the U.S., Europe, and Japan for patients with T2DM; however, analogous evidence is lacking for Saudi T2DM patients. Therefore, we assessed the efficacy and safety of empagliflozin as an add-on to ongoing antidiabetic therapy in Saudi adult patients with T2DM in the Armed Forces Hospital, Southern Region between June and December 2021.

## 2. Materials and Methods

### 2.1. Study Design and Patients

This was a prospective, open-label, observational clinical study conducted at the Endocrine and Diabetes Centre of the Armed Forces Hospital, Southern Region (Saudi Arabia) between June and December 2021. The study protocol was approved by the Armed Forces Hospital, Southern Region Research Ethics Committee (Number: AFHSRMREC/2021/PHARMACY/503). All participants provided signed and dated informed consent prior to screening.

Saudi adult participants aged ≥18 to <80 years were screened to determine whether they met the inclusion criteria: Adult Saudi patients with T2DM in the Endocrine and Diabetes Centre of the Armed Forces Hospital, Southern Region in Saudi Arabia. Patients who had not been treated with EMPA before enrolment were eligible. The exclusion criteria included T2DM patients less than 18 years of age, adults with type one diabetes, and pregnant women. The study population had no cardiovascular or renal disease.

This study was designed to assess the safety and efficacy of EMPA in Saudi adult patients with T2DM. Efficacy and safety analyses were based on a comparison between variables before treatment (baseline group) and in the patients treated with EMPA (25 mg/once daily) for 12 weeks (EMPA group). Efficacy measurements included changes from baseline in HbA1c levels, fasting plasma glucose (FPG) and body weight at week 12. Safety assessments included changes in BP (SBP and DBP), pulse rate, kidney markers (urine albumin/creatinine ratio (UACR) and estimated glomerular filtration rate (eGFR)), liver markers (AST, ALT, and GGT), and serum lipids (LDL-c, HDL-c, and TG) at week 12 [39].

### 2.2. Patient Demographics at Baseline

Sample size calculations were based on a previous study of empagliflozin with insulin, which suggested that empagliflozin would result in an HbA1c reduction of ~0.5% versus placebo after 12 weeks of treatment, and a standard deviation (SD) of 1.0% [33]. The sample size was 256 patients (113 males and 234 females) with the following characteristics: mean age 58.9 years; males 58.2 and females 59.4. Of 256 patients, 234 (91%) had been diagnosed with T2DM longer than five years, and 22 (9%) had been diagnosed for one to five years. Participating patients had insufficient glycaemic control at baseline, with HbA1c levels ≥7% in 251 patients (97.6%). Further, 167 patients (65%) had HbA1c levels ≥9 despite receiving insulin (156, 64%) or OHA (93, 36%) (Table 1).

### 2.3. Data Analysis

SPSS (Version 27.0, IBM, Armonk, NY, USA) was employed for statistical analysis using the *t*-test for two independent samples to determine the rate of change in the means of the two samples, standard deviations, and the level of confidence, which is estimated at 0.05.

Confidence domain: If the level of significance required by researchers is 5%, then the confidence level should be 95%. Thus, the confidence interval contains the possible values of the statistical parameter, which, when subjected to a statistical test using the same sample, will not be rejected. The level of statistical significance for all samples studied was less than 5%, which means that all rates of change in the mean were within the confidence range.

## 3. Results

### 3.1. Efficacy

The primary efficacy endpoints were changes in HbA1c, FPG, and body weight from baseline at week 12 (EMPA group). The mean value of HbA1c decreased from 9.77 ± 1.76 to 8.85 ±4.83 with a change of −0.93 (−0.32, −1.54) at a rate of (−0.106) within the confidence interval estimated CI at 95%. In addition, 181 participants (72%) experienced a reduction in HbA1c ≥ 0.5% from baseline. Further, the number of participants with HbA1c ≥ 9% decreased from 167 (65%) to 77 (30%), corresponding to an increase in the proportion of individuals with HbA1c levels ≥8% to <9%, ≥7% to <8%, and <7.0% of (80 to 82), (4 to 76), and (5 to 21), respectively. For participants on insulin and OHA (metformin) therapies (156, 64%), there was a reduction in mean HbA1c % from 9.94 ± 1.84 to 8.66 ±1.47, a change of −1.28 (−1.03, −1.53) at a rate −0.147, accompanied by a reduction in insulin units from 9.94 ± 1.84 to 8.67 ± 1.47 (change −1.27). Meanwhile, for those on OHA only, the reduction in HBA1c % was from 9.46 ± 1.56 to 8.31 ± 1.26 with a change of −1.11 (−0.799, −1.42).

**Figure 1:**
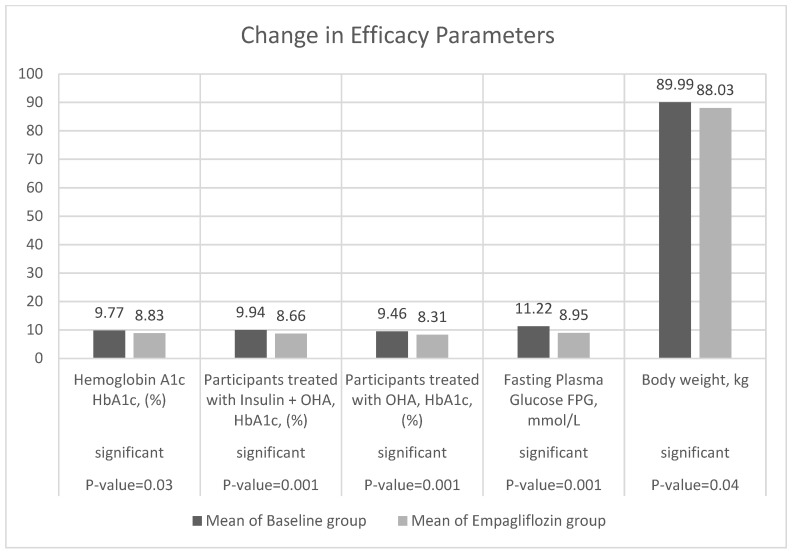
Change from baseline in HbA1c%, FPG mmol/L, and body weight kg at week 12 (*t*-test).

The mean value of FPG reduced from 11.22 ± 4.79 to 8.95 ±3.37, a change of −2.28 (−2.81, −1.75) at a rate of (−0.25) and a 95% CI. A total of 63 participants had FPG levels <7.8 at baseline increased to 115 at week 12. Individuals with FPG levels from 7.8 to <11.0 (80 to 86), while the number with FPG levels ≥11 decreased from 113 to 55. Mean body weight decreased from 89.99 ± 18.09 to 88.03 ± 18.47 with a change of −0.874 (−4.36, −6.10) at a rate of −0.02 (Table 2 and Table 3 and Figure 1).

### 3.2. Safety

**Figure 2:**
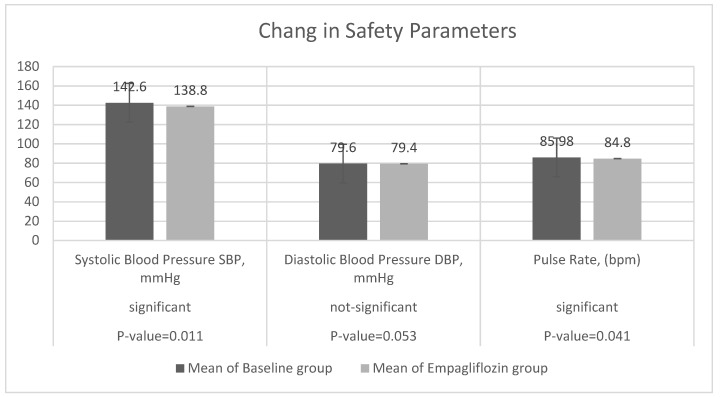
Change from baseline in SBP mmHg, DBP mmHg, and pulse rate bpm at week 12 (*t*-test).

Safety endpoints included changes in SBP, pulse rate, and kidney and liver function status at week 12. SBP decreased from 142.6 ± 19.45 to 138.8 ± 20.23, a change of −3.85 (−6.81, −0.88) at a rate of −0.03. There was a slight reduction in DBP from 79.6 ± 20.32 to 79.4 ± 21.14, a change of −0.06 (−0.81, −0.88) at a rate of −0.001. Moreover, there was a reduction in pulse rate from 85.98 ± 11.33 to 84.80 ± 13.52, a change of −1.18 (−0.79, −3.15) at a rate of −0.01 (Table 2 and Table 3 and Figure 2).

**Figure 3:**
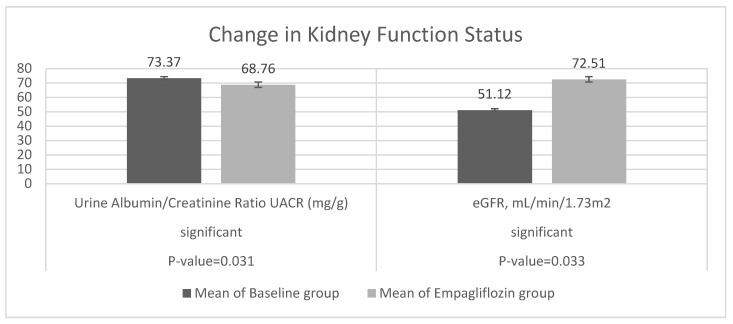
Change from baseline in UACR mg/g and eGFR mL/min/1.73 m^2^ at week 12 (*t*-test).

It is apparent from Table 2 and Table 3 that kidney function status improved, with a reduction in UACR from 20.39 ± 43.72 to 17.12 ± 40.05 at week 12, a change of −1.76 (−1.07, −34.25) at a rate of −0.067. The number of participants with UACR levels ≥300 decreased from 75 to 8, accompanied by an increase in the number of patients with UACR levels of 30 to <300 and <30 (from 51 to 67 and 126 to 177 patients, respectively). Furthermore, estimated eGFR also increased from 51.12 ± 120.45 to 72.51 ± 22.80, a change of 3.54 (2.78, 9.87) at a rate of 0.418. The number of participants with normal eGFR (≥90) increased from 49 to 55, along with a reduction in the number of participants with mild eGFR (60 to < 90; from 120 to 116 patients) and moderate-to-severe eGFR (30 to < 60; from 86 to 84 patients). For one participant with severely decreased eGFR (<30), no change was observed (Figure 3).

**Figure 4:**
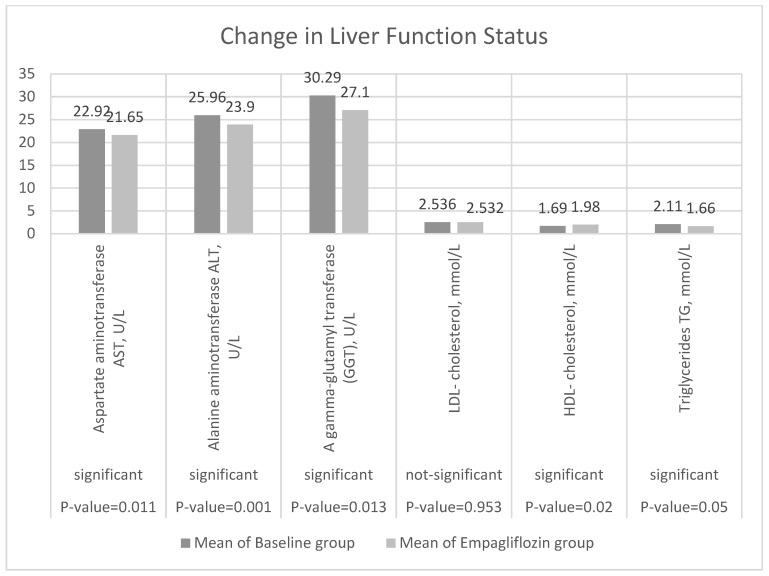
Change from baseline in AST U/l, ALT U/L, GGT U/L, LDL mmol/L, HDL-c mmol/L, and TG mmol/L at week 12 (*t*-test).

The results shown in Table 2 and Table 3 show the rates of change in hepatic function status between the baseline and EMPA groups. AST decreased from 22.92 ± 8.10 to 21.65 ± 6.38, a change of −1.26 (−0.3011, −2.227) at a rate of −0.06. ALT decreased from 25.96 ± 8.09 to 23.91 ± 11.71, a change of −2.36 (−1.03, −3.69) at a rate (−0.029). GGT decreased from 30.29 ± 25.15 to 27.12 ± 18.32, a change of −4.31 (−2.33, −6.28) at a rate of −0.159. Interestingly, lipid profiles improved as well (Table 2 and Table 3). HDL increased significantly from 1.69 ± 3.59 to 1.98 ± 0.22, a change of 0.29 (0.74, 0.15) at a rate of 0.171, while LDL increased non-significantly from 2.543 ± 0.93 to 2.544 ±1.50, a change of 0.005 (0.192, 0.18) at a very small rate of 0.0004. Additionally, TG levels decreased from 2.11 ± 5.90 to 1.66 ± 0.20, a change of −0.43 (−0.31, −1.17) at a rate of −0.271 (Figure 4).

## 4. Discussion

The present study was designed to determine the efficacy and safety of EMPA for Saudi T2DM patients. In this 12-week study, once-daily add-on administration of EMPA (25 mg) resulted in significant decreases in HbA1c, FPG, and body weight for Saudi participants with insufficiently controlled T2DM on insulin or OHA. The meaningful improvement in HbA1c was seen in the participants with HbA1c ≥ 9.0, representing 65% of individuals at baseline group. Furthermore, after 12 weeks of treatment, a decrease in HbA1c ≥ 0.5% from baseline was recorded in 239 (93%) of the participants receiving 25 mg of EMPA. These findings are in line with those of another study that found that 12-week treatment with EMPA monotherapy resulted in similar reductions in HbA1c, FPG, and body weight compared to a placebo in drug-free patients with T2DM [40]. Reductions in HbA1c, FPG, and body weight were also consistent with those reported from 12-week studies on other SGLT2 inhibitors [33,41,42]. EMPA as an added therapy to insulin resulted in a significant reduction in insulin units, consistent with the results reported by [43]. Control of blood glucose levels is important in diabetic patients but often associated with weight gain [44]. The potential for a reduction in body weight is a notable feature of SGLT2 inhibitors [45,46] and may make them useful agents to combine with other antidiabetic therapies to reduce glucose levels and facilitate weight loss or mitigate any weight gain associated with improved glycaemic control [13]. Caloric loss through urinary glucose excretion may be an important contributor to this effect [22].

The reduction in BP observed in the current study is consistent with a reduction in SBP reported with a 25 mg dose of EMPA [47]. It is conceivable that EMPA stimulates osmotic diuresis through increased glycosuria rather than natriuresis, which may play a role in the potential antihypertensive effects of EMPA [48] and support its mechanism as an inhibitor of the renin–angiotensin system [49]. In addition, in Black individuals with T2DM, EMPA reduced BP although the full antihypertensive effect took ≥ 6 months to be fully realised [50]. Moreover, the cardioprotective mechanism of SGLT2 is reported [51]. The hypotheses on SGLT2 mechanisms of action have changed: from simple glycosuric drugs, with consequent glucose lowering, erythropoiesis enhancing, and ketogenesis stimulating, to intracellular sodium-lowering molecules. This provides their consequent cardioprotective effect, which justifies its significant reduction in CV events, especially in populations at higher risk [51]. Hyperglycaemia is a well-established cause of endothelial dysfunction (ED) in the pathophysiology of diabetic complications This abnormal vascular phenotype represents an important risk factor for the genesis of any complication of diabetes, contributing to the pathogenesis of not only macrovascular disease but also microvascular damage. Gliflozins have cardiovascular protective mechanisms of SGLT2 inhibition in patients T2DM and their impact on endothelial function [52].

The present study demonstrated an improvement in kidney function, reduced UCAR, and improved eGFR. Diabetes-associated kidney disease is the most common cause of end-stage renal disease in most countries. Diabetic kidney disease, which develops in approximately 40% of patients with T2DM, further increases the risk of cardio-vascular-related morbidity and mortality [53]. Reported kidney protection with EMPA supports our results [25,54]. It has also been shown that EMPA has a beneficial effect on key efficacy outcomes and slows the rate of kidney function decline in patients with and without chronic kidney disease CKD, regardless of the severity of kidney impairment at baseline [25]. The renal-protective effects of EMPA are likely due to a combination of several mechanisms, including EMPA-associated body weight and BP reductions, diuresis, a shift in substrate utilisation, and activation of tubuloglomerular feedback [53]. In addition, haemodynamic effects of EMPA, associated with a reduction in intraglomerular pressure, may contribute to long-term preservation of kidney function [28]. The fact that SGLT2 inhibition is also associated with small decreases in eGFR over the first 3–4 weeks’ treatment suggests that reductions in intraglomerular pressure associated with EMPA may further contribute to the UCAR-lowering effects [55].

Mechanistic insights suggest that ectopic liver fat is probably part of the pathogenic process in diabetes, contributing to hepatic insulin resistance, excess gluconeogenesis, and higher fasting glucose levels [56]. Furthermore, hepatic steatosis due to non-alcoholic fatty liver disease (NAFLD) leads to and is often clinically suspected by increased levels of aminotransferases, with levels of alanine aminotransferase (ALT) exceeding those of aspartate aminotransferase (AST). Elevated ALT levels (typically >40–50 U/l) are common in individuals with type 2 diabetes and for a given serum ALT, and those with type 2 diabetes have more liver fat compared with BMI-, age-, and sex-matched individuals without diabetes [30]. The mechanisms by which empagliflozin might reduce aminotransferases or liver fat are unclear. Data from animal models support a direct effect of empagliflozin on reducing liver fat and improving hepatic glucose handling. In db/db mice, glucose uptake in the liver and kidneys has been reported to be higher in mice treated with empagliflozin than in controls [57]

In this study, EMPA was found to elicit reductions in aminotransferases ALT, AST, and GGT in Saudi patients with T2DM, and the reduction in ALT was greater than the reduction in AST. These results match those observed in an earlier study [58]. This pattern is consistent with a reduction in liver fat, especially when ALT levels are high [30].

One of the most important clinically relevant findings of the current study was the reduction in plasma TG levels and the increase in HDL-c but not LDL-c levels. These findings are in agreement with [58]. The mechanism behind this finding was also reported. Specifically, EMPA increases serum campesterol, a marker of cholesterol absorption, in patients with T2DM. This increase may be associated with SGLT2 inhibitor-induced increases in HDL cholesterol [59]. Further research is needed to investigate the adverse effects of EMPA on Saudi population with T2DM, which have not yet been documented.

## 5. Conclusions

This is the first study reporting the efficacy and safety of EMPA as an add-on to antidiabetic therapy (insulin or oral hypoglycaemic agent) in Saudi patients with T2DM.

## Figures and Tables

**Table 1:** Patient demographics.

Patient Demographics	
Sample volume, *n*	256
Age (years), mean (SD)	58.9 (10.75)
Sex Males, *n*	113
Females, *n*	143
Male age, mean (SD)	58.2 (11.88)
Female age, mean (SD)	59.4 (9.76)
**Duration since Diagnosis of T2DM, (years)**	
Mean (SD)	16.7 (8.47)
<1, *n* (%)	0
1 to 5	22 (9%)
> 5	234 (91%)
**DM Treatment before Empagliflozin**	
Insulin + OHA (metformin)	156 (64%)
OHA (metformin)	93 (36%)

SD: Standard deviation, T2DM: Type 2 diabetes mellitus, DM: Diabetes mellitus, OHA: Orally administered antihyperglycemic.

**Table 2:** Efficacy and Safety Parameters at baseline and week 12.

	Baseline Group	Empagliflozin Group	Change Amount
	Mean (SD)	Mean (SD)	
**Efficacy:**			
Haemoglobin A1c HbA1c, (%)	9.77 ± 1.76	8.85 ± 4.83	−0.92
Participants treated with Insulin + OHA, HbA1c, (%)	9.94 ± 1.84	8.66 ± 1.47	−1.27
Participants treated with	9.46 ± 1.56	8.31 ± 1.26	−1.15
OHA, HbA1c, (%)			
Fasting Plasma Glucose FPG, mmol/L	11.27 ± 4.79	8.95 ± 3.37	−2.32
Body weight, kg	89.99 ± 18.09	88.03 ± 18.47	−1.96
**Safety:**			
Systolic Blood Pressure SBP, mmHg	142.6 ± 19.45	138.8 ± 20.23	−3.8
Diastolic Blood Pressure DBP, mmHg	79.6 ± 20.32	79.4 ± 21.14	−0.2
Pulse Rate, (bpm)	85.98 ± 11.33	84.80 ± 13.52	−1.18
**Kidney Function Status**			
Urine Albumin/Creatinine Ratio UACR (mg/g)	20.39 ± 43.72	17.12 ± 40.05	−3.27
eGFR, mL/min/1.73 m^2^	51.12 ± 120.45	72.51 ± 22.80	21.39
**Liver Function Status**			
Aspartate aminotransferase AST, U/L	22.92 ± 8.10	21.65 ± 6.38	−1.26
Alanine aminotransferase ALT, U/L	25.96 ± 8.09	23.91 ± 11.71	−0.70
A gamma-glutamyl transferase (GGT), U/L	30.29 ± 25.15	27.12 ± 18.32	−4.31
LDL- cholesterol, mmol/L	2.543 ± 0.93	2.544 ± 1.50	0.0009
HDL- cholesterol, mmol/L	1.69 ± 3.59	1.98 ± 0.22	0.29
Triglycerides TG, mmol/L	2.11 ± 5.90	1.66 ± 0.20	−0.45

HbA1c: haemoglobin A1c (glycosylated haemoglobin), FPG: fasting plasma glucose, SD: standard deviation, OHA: Orally administered antihyperglycemic, eGFR: Estimated glomerular filtration rate, LDL: Low-density lipoprotein, HDL: High-density lipoprotein, AST: Aspartate aminotransferase, ALT: Alanine transaminase and TG: Triglycerides. A comparison between Empagliflozin-treated group at week 12 with the same group at bassline. Data are *n* (%) or mean (SD).

**Table 3:** Change in Efficacy and Safety Parameters at week 12.

	Baseline Start Treatment Empagliflozin	Change from Baseline at Week 12	Rate of Change
**Efficacy variables**			
Mean change in HbA1c	−0.93 (−0.32, −1.54)		−0.106
from baseline, % (95% CI)			
Mean ± SD	−0.93 ± 4.93		
<7.0%	5 (0.02)	21 (0.08)	0.76
≥7% to <8%	4 (0.016)	76 (0.30)	0.95
≥8% to <9%	80 (0.31)	82 (0.32)	0.02
≥9%	167 (0.65)	77 (0.30)	−0.54
Decrease in HbA1c (%) in participants: ≥ 0.5%, *n*, (%)	181(0.71)		
Treated with insulin + OHA, HbA1c, (%)	−1.28(−1.03, −1.53)		−0.147
Treated with OHA, HbA1c (%)	−1.11(−0.799, −1.42)		−0.139
Decrease in insulin units in participants treated with insulin + OHA	9.94 ± 1.84	8.67 ± 1.47	−1.27
Mean change in FPG	−2.28 (−2.81, −1.75)		−0.25
from baseline (95% CI)			
Mean ± SD	−2.27 ± 4.22		
<7.8, *n*, (%)	63 (0.25)	115 (0.45)	0.83
7.8 to <11.0	80 (0.32)	86 (0.34)	0.08
≥11	113 (0.44)	55 (0.21)	−0.51
Mean change in body weight	−0.874 (−4.36, −6.10)		−0.02
from baseline (95% CI)			
Mean ± SD	−1.96 ± 11.98		
**Safety variables**			
Mean change in SBP	−3.85 (−6.81, −0.88)		−0.03
from baseline, % (95% CI)			
Mean ± SD	−3.82 ± 23.4		
Mean change in DBP	−0.06 (−0.81, −0.88)		−0.001
from baseline, % (95% CI)			
Mean ± SD	−0.05 ± 0.28		
Mean change in pulse rate	−1.18 (−0.79, −3.15)		−0.01
from baseline (95% CI)			
Mean ± SD	−1.18 ± 15.46		
**Kidney Function Status**			
Mean change in Urine Albumin/			−0.067
Creatinine Ratio UACR (mg/g)	−1.76 (−1.07, −34.25)		
Mean ± SD	−1.76 ± 103.5		
<30, *n*, (%)	126 (0.50)	177(0.70)	0.4
30 to <300 *n*, (%)	51(0.21)	67 (0.27)	0.31
≥300, *n*, (%)	75 (0.29)	8 (0.03)	−0.89
Mean change in body eGFR	3.54 (2.78, 9.87)		−0.418
from baseline, kg (95% CI)			
Mean ± SD	3.54 ± 45.98		
≥90, *n*, (%)	49 (0.19)	55 (0.21)	0.02
60 to < 90	120 (0.47)	116 (0.45)	−0.018
30 to < 60	87 (0.34)	84 (0.33)	−0.016
**Liver Function Status**			
Mean change in AST	−1.26 (−0.3011, −2.227)		−0.058
from baseline (95% CI)			
Mean ± SD	−1.263 ± 7.72		
Mean change in ALT	−2.36 (−1.031, −3.69)		−0.029
from baseline (95% CI)			
Mean ± SD	−2.36 ± 10.75		
Mean change in GGT	−4.31 (−2.33, −6.28)		−0.159
from baseline (95% CI)			
Mean ± SD	−4.31 ± 15.66		
Mean change in LDL	0.005 (0.192, 0.18)		0.0004
from baseline (95% CI)			
Mean ± SD	0.005 ± 1.46		
Mean change in HDL	0.29 (0.74, 0.15)		0.171
from baseline, kg (95% CI)			
Mean ± SD	0.293 ± 3.61		
Mean change in TG	−0.43 (−0.31, −1.17)		−0.271
from baseline (95% CI)			
Mean ± SD	−0.45 ± 5.98		

A comparison between EMPA treated group at week 12 with the same group at baseline. Data are *n* (%) or mean (SD).

## Data Availability

The data that support the findings of this study are openly available from the corresponding author upon reasonable request.
